# Clinical and *in vitro* Study of Novel Long Non-Coding RNA lncUSMycN in Breast Cancer

**DOI:** 10.29252/.23.5.303

**Published:** 2019-09

**Authors:** Reyhaneh Ravanbakhsh Gavgani, Esmaeil Babaei, Mohammad Ali Hosseinpourfeizi, Ashraf Fakhrjou, Vahid Montazeri

**Affiliations:** 1 Department of Biological Sciences, School of Natural Sciences, University of Tabriz, Tabriz, Iran;; 2 Department of Pathology, Faculty of Medicine, Tabriz University of Medical Sciences, Tabriz, Iran;; 3 Department of Thoracic Surgery, Noor-Nejat Hospital, Tabriz, Iran

**Keywords:** Breast cancer, Real-time polymerase chain reaction, siRNA

## Abstract

**Background:**

Despite recent advances in diagnosis and treatment, breast cancer remains a leading cause of death in women worldwide. Long non-coding RNAs are a new class of RNA molecules that have been shown to participate in tumorigenesis. The aim of this study was to investigate the expression of lncUSMycN in tumor samples and to evaluate its potential role in the breast cancer cell line.

**Methods:**

Real-time polymerase chain reaction was employed to assess lncUSMycN expression in breast tumor tissues and cancer cell lines. Furthermore, small interfering RNA was used to knockdown lncUSMycN.

**Results:**

The data showed the significant up-regulation of lncUSMycN in tumor tissues compared to non-tumor specimens (95% CI, *p* = 0.002). Receiver operating characteristic (ROC) curve analysis demonstrated the biomarker potential of lncUSMycN (ROCAUC = 0.70, *p* < 0.001) for invasive breast ductal carcinoma. Furthermore, lncUSMycN knockdown induced apoptosis and suppressed cellular migration in breast cancer cells (*p* < 0.01).

**Conclusion:**

The findings highlight the pivotal role of lncUSMycN in tumorigenesis, providing a new potential target for breast cancer therapy.

## INTRODUCTION

Breast cancer is the most common type of malignancy and the second leading cause of cancer-related death in women worldwide^[^^1^^,^^2^^]^. Improving diagnosis and treatment strategies in early stages of the disease remains a high priority; thus, a deeper understanding of the molecular and genetic networks that control tumor initiation and progression is crucial.

With the advent of new transcriptome sequencing technologies, it has been determined that almost 90% of the human genome is actively transcribed into non-coding RNAs^[^^3^^]^. Long non-coding RNAs (lncRNAs) are endogenous cellular RNA molecules longer than 200 nucleotides in length, which have lately received a great attention as important regulators of gene expression^[^^4^^]^. 

Increasing evidence has shown that lncRNAs can either directly or indirectly affect various cellular pathways, including those associated with cell survival, apoptosis, and metastasis; thus, aberrant expression of lncRNAs can adversely affect a wide range of biological processes resulting in different diseases, including cancer. lncRNAs such as MEG3, HOTAIR, and HULC have been demonstrated to be involved in tumorigenesis, tumor progression, and invasion^[^^5^^,^^6^^]^. These findings further emphasize the potential of lncRNAs as suitable diagnostic and/or prognostic biomarkers as well as novel targets in cancer therapy. In 2014, a lncRNA, denoted as long non-coding upstream MycN (lncUSMycN), was firstly reported in patients with neuroblastoma^[^^7^^]^. *LncUSMycN* gene is located on chromosome 2p15.9, 14-kbp upstream of the MYCN transcription start site. A preliminary report has shown that the elevated expression of lncUSMycN is associated with a poor prognosis in neuroblastoma patients. In addition, lncUSMycN has a regulatory role in cell proliferation and tumorigenesis of neuroblastoma^[^^7^^]^. However, little data on the expression and function of lncUSMycN in cancer exist; thus, we aimed to study the expression profile of lncUSMycN in invasive ductal carcinoma specimens in comparison with non-tumoral tissues. Moreover, siRNA-mediated knockdown was used to investigate the role of lncUSMycN in breast cancer cells.

## MATERIALS AND METHODS

**Human tissue specimens**

Fifty-two specimens of surgically resected breast tumors with the invasive ductal carcinoma subtype and their adjacent non-tumor tissues (no obvious tumor cells were detectable; as evaluated by an expert pathologist) were collected from Noor-Nejat and Emam Reza Hospitals (Tabriz, Iran). The age of the patients ranged from 34 to 80 years (average: 51.29 ± 1.73). A written consent was obtained from each patient prior to sample collection, and the study was carried out with the approval of the Ethical Committee of Tabriz University of Medical Sciences (approval number: IR.TBZMED.REC.1392.249). The specimens were immediately frozen in liquid nitrogen after surgery and stored at -80 °C. The TNM staging was carried out according to the American Joint Committee on Cancer (AJCC; 8^th^ edition).

**Cell culture**

All human breast cancer cell lines, including ZR-75-1 (ATCC NO. CRL-1500), MCF-7 (ATCC No. HTB-22), and MDA-MB-231 (ATCC NO. HTB-26) were obtained from the National Cell Bank of Pasteur Institute of Iran, Tehran. ZR-75-1 and MCF-7 were grown in RPMI-1640 (Gibco, Life Technologies, Carlsbad, CA, USA), while MDA-MB-231 was cultured in DMEM (Sigma-Aldrich, St. Louis, MO, USA) supplemented with 10% heat-inactivated FBS (Gibco) and 100 U/ml of penicillin/streptomycin (10.000 U/mL, Gibco) in a humidified atmosphere of 5% CO_2_ at 37 °C.

**RNA extraction, cDNA synthesis, and quantitative RT-PCR (qRT-PCR)**

Total RNAs were extracted using TRIzol reagent (Invitrogen, Carlsbad, CA, USA) according to the manufacturer’s instructions. cDNA was synthesized by reverse transcription of 1 μg of total RNA using the PrimeScript RT kit (Takara, China) by following the manufacturer's protocol. qRT-PCR was carried out with primers specific for LncUSMycN, Bax, Bcl-2, E-cadherin, MMP-9, and β-actin using SYBR® Premix EX TaqTM II (Tli RNaseH Plus; Takara) by Corbett Rotor-Gene 6000 (Corbett Life Sciences, Germany). Primer sequences and qRT-PCR conditions are outlined in Table 1. All gene expression levels were normalized to β-actin as reference.

**PCR product sequencing**

To further confirm the expression of lncUSMycN, RT-PCR products were subjected to Sanger sequencing, subsequently interpreted by Chromas Pro 2.4.1 and aligned in BLAST. Primers used for sequencing were as forward: 5'-GTGTGTCTGTTGCT GAATG-3' and reverse: 5'-CTGTGTGTGCTTTGG TGCT-3'.

**siRNA transfection**

To investigate the role of lncUSMycN, its expression was suppressed via siRNA-mediated gene silencing. Therefore, pre-designed negative control siRNA (si-NC) and custom-synthesized si-lncUSMycN against lncUSMycN (target sequence: 5′-GGCAGGGAAGGT GTTGTTGTT-3′) were purchased from Dharmacon (Lafayette, CO, USA). Both siRNAs were labeled with 6-Carboxyfluorescein (6-FAM) at their 3' end to measure them during *in vitro* experiments. Cells were transfected using HiPerFect Transfection Reagent (Qiagen, Germany) according to the manufacturer’s protocol. Assays were performed 72 h after transfection.

**Study of apoptosis**

To study apoptosis and necrosis, acridine orange/ethidium bromide staining was employed. Briefly, ZR-75-1 cells (2.5 10^5^) were seeded onto 24-well plates and cultured for 24 h. Then the cells were transfected with si-lncUSMycN and si-NC separately according to the manufacturer's instruction. After 72 h, the cells were detached by Trypsin-EDTA (0.25%; HyClone; GE Healthcare Life Sciences, USA) and transferred to glass slides, followed by staining with acridine orange/ethidium bromideAO/EtBr solution containing 100 µg/ml acridine orange and 100 µg/ml ethidium bromide (Sigma-Aldrich). Finally, the cells were analyzed under fluorescence microscope (Olympus BX 41, Germany). In addition, the expression of apoptosis-related genes *Bax* and *Bcl-2* was evaluated by qRT-PCR.

**Table 1:** The sequences of the primers used in this study and their qPCR programs

**Primer name**	**Primer sequence (5'-3')**	**qPCR cycling ** **program** * ** min (s)**	**Product ** **size (bp)**
LncUSMycN-F LncUSMycN-R	ACTTGTCCTGCGTGCTTGTT TGTGTGTGCTTTGGTGCTCA	D: 95 ( 30) A: 57 (22) E: 72 (20)	218
Bax-F Bax-R	GCAAACTGGTGCTCAAGG ACTCCCGCCACAAAGA	D: 95 (30) A: 63 (35) E: 72 (30)	236
BCL2-F BCL2-R	TGGGAAGTTTCAAATCAGC GCATTCTTGGACGAGGG	D: 95 (25) A: 63 (30) E: 72 (30)	298
E-cadherin-F E-cadherin-R	AGTACAACGACCCAACCCAAG GCAAGAATTCCTCCAAGAATCC	D: 95 (30) A: 57 (22) E: 72 (20)	235
MMP-9-F MMP-9-R	CCGCTCACCTTCACTCGC ACCACAACTCGTCATCGTC	D: 95 (30) A: 63 (35) E: 72 (30)	174
β-actin-F β-actin-R	AGAGCTACGAGCTGCCTGAC AGCACTGTGTTGGCGTACAG		184

* qPCR was started for all primers with initial denaturation at 95 °C for 10 min; D, denaturation; A, primer annealing; E, extension**; **F, forward; R, Reverse

**Cell cycle analysis**

To perform cell cycle analysis, ZR-75-1 cells (2.5 X10^5^) were seeded in 24-well plates for 24 h, followed by transfection with si-lncUSMycN and si-NC separately according to manufacturer's instructions. After 72 h, the cells were detached by Trypsin-EDTA solution at 37 °C for 5 min, followed by adding 10% FBS-RPMI 1640 medium, in order to inhibit Trypsin activity. Afterward, the cells were washed by cold PBS, followed by fixation using ice-cold ethanol (70% w/w). Next, the cells were incubated in a freshly prepared solution containing 0.1% Triton X-100, RNase A (50 μg/ml; Sigma-Aldrich), and propidium iodide (PI) (50 μg/ml; Sigma-Aldrich) at 4 °C for 15 min. Then the stained cells were analyzed by flow cytometry (BD FACS Calibur flow cytometer, BD Biosciences, USA). The percentage of cells in the subG1 and G1 phases were analyzed by FlowJO 7.6.1 software. 

**Wound healing assay**

In order to get around 90% confluency after 24 h of culturing, ZR-75-1 cells were seeded at appropriate numbers into a 24-well plate in complete medium. After 24 h, a single wound was created in the middle of the well using a sterile 200 μl pipette tip. After removing detached cells with PBS (phosphate buffered saline), the adherent cellular layers were separately transfected with si-lncUSMycN and si-NC according to the manufacturer's instructions. After 72 h, the cells migrated into the wounded area were photographed under an inverted microscope (Olympus, Japan). Beside wound healing assay, the expression of genes implicated in invasion and metastasis, including *CDH1* (E-cadherin) and *MMP-9*, was assessed at mRNA levels. 

**Statistical analysis**

Relative Expression Software Tool (REST) 2009 was used to evaluate the statistical differences in lncUSMycN expression of the breast cancer tissues relative to their non-tumoral counterparts. The fold change and relative expression of lncUSMycN were calculated by the 2^−ΔΔCt^ (ΔΔCt = ΔCt [treated cells] - ΔCt [control]) and 2^-ΔCt^ (ΔCt = Ct [lncUSMycN] - Ct [β-actin]) methods, respectively. Also, Student’s *t*-test and one-way ANOVA were performed to compare two or more groups, respectively. *p* values < 0.05 were considered to be statistically significant. Receiver operating characteristic (ROC) curve analysis was plotted to assess the biomarker potential of lncUSMycN in breast cancer. All experiments were repeated at least three times, and data were represented as mean ± SEM (standard error of mean).

## RESULTS

**LncUSMycN is up-regulated in breast cancer tissues**

**Fig. 1:**
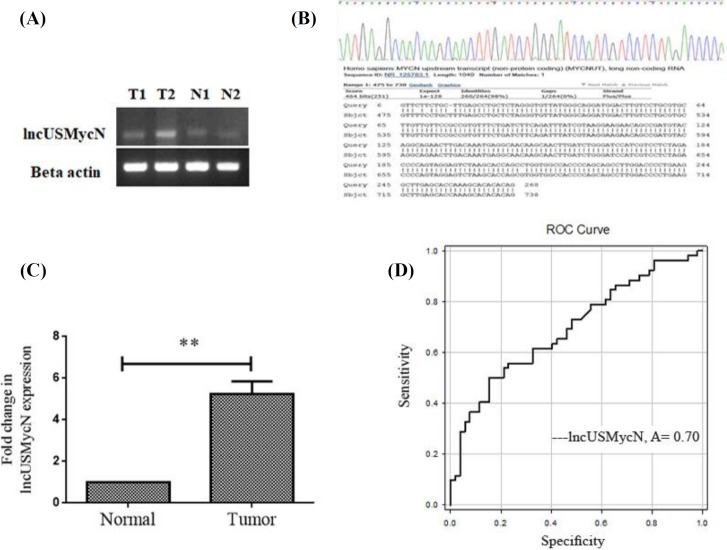
Expression of lncUSMycN in breast cancer and receive operating characteristic (ROC) curve analysis for prediction of breast cancer prognosis. (A) Amplified lncUSMycN and β-actin on 2% agarose gel; (B) confirmation of lncUSMycN expression by Sanger sequencing; (C) qRT-PCR analysis of lncUSMycN expression level in cancerous and adjacent non-cancerous breast tissues. lncUSMycN was up-regulated in tumor tissues compared to adjacent normal tissues; (D) ROC curve for lncUSMycN to discriminate between tumor and normal tissues. The area under the ROC curve (AUC) was 0.70 (*p *< 0.001). The sensitivity and specificity were 0.85 and 0.55, respectively, and cut-off value was 17.5 (^**^p < 0.01)

To study the role of lncUSMycN in breast cancer, lncUSMycN expression was first detected in breast tumor tissues. As Figure 1A shows, the PCR amplicon for lncUSMycN is specifically synthesized by primers. The PCR product was validated by Sanger sequencing (Fig. 1B). The results for expression studies showed that lncUSMycN is significantly up-regulated by 5.2 fold in cancerous tissues compared to non-cancerous specimens (95% CI, 5.241 ± 0.355, *p *= 0.002, Fig. 1C). Furthermore, it was found that lncUSMycN expression was significantly correlated with early stages of breast cancer (*p* = 0.004). No association was detected between expression of lncUSMycN and other clinicopathological features, including age, lymph node metastasis, differentiation, tumour location and progesterone, estrogen, and HER-2 receptor levels (Table 2). ROC curve analysis was done to determine whether or not cancerous and non-cancerous tissue could be distinguished by the expression level of lncUSMycN. The area under the ROC curve (AUC) was 0.70 (*p *< 0.001). The sensitivity and specificity were 0.85 and 0.55, respectively, and the cut-off value was 17.5 (Fig. 1D).

**lncUSMycN expressed in breast cancer cell lines**

**Fig. 2:**
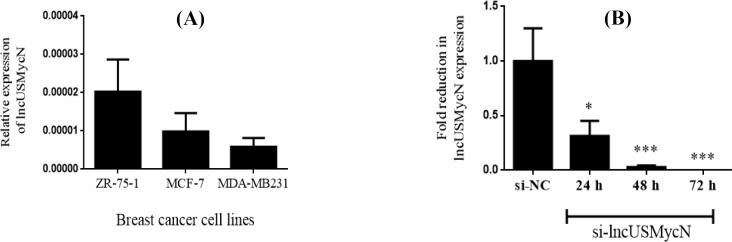
lncUSMycN expression in breast cancer cell lines and siRNA-mediated knockdown. (A) LncUSMycN expression levels in breast cancer cell lines, including ZR-75-1, MCF-7, and MDA-MB-231; (B) the efficiency of siRNA-mediated knockdown evaluated 24 h, 48 h, and 72 h after transfection by RT-qPCR in ZR-75-1 cells. ^*^p < 0.05, ^***^p < 0.001. si-NC, siRNA negative control; si-lncUSMycN, siRNA lncUSMycN

To further evaluate the potential role of lncUSMycN in breast cancer, siRNA-based knockdown was employed in ZR-75-1, MCF-7, and MDA-MB-231 cell lines. However, due to the inherent higher expression of lncUSMycN in ZR-75-1, the data for this cell line are illustrated in Figure 2A. qRT-PCR data revealed that lncUSMycN was significantly knocked down in siRNA-treated cells by more than 90% efficiency. Fold reduction in lncUSMycN expression following si-lncUSMycN transfection was significant (*p *< 0.001, Fig. 2B).

**lncUSMycN knockdown induced apoptosis**

**Fig. 3:**
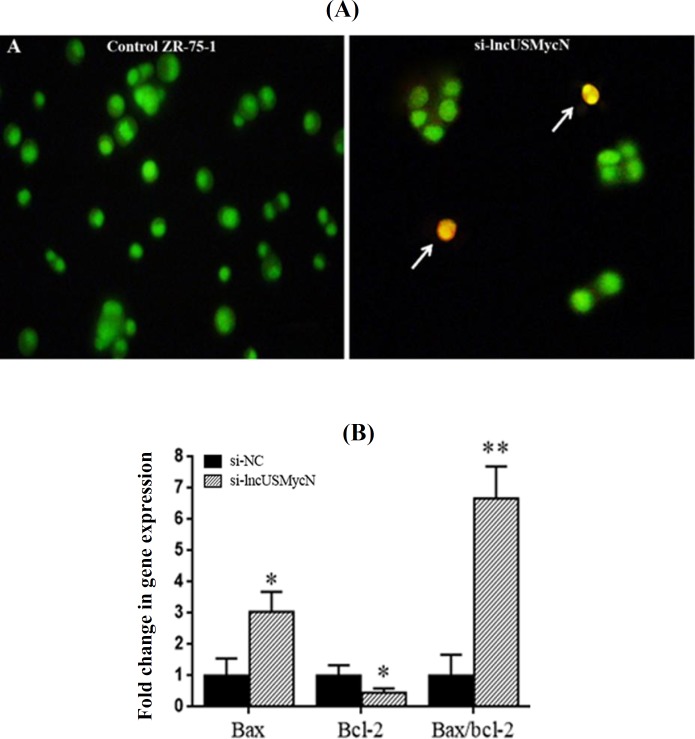
Effects of lncUSMycN knockdown on apoptosis. (A) Morphological evaluation of the apoptotic cells stained with acridine orange/ethidium bromide (AO/EtBr). Arrows indicate apoptotic cells. (magnification 200 ); (B) expression of Bax and Bcl-2 in ZR-75-1 cells by qRT-PCR 72 after lncUSMycN knockdown. ^*^p < 0.05, ^**^p < 0.01. si-NC, siRNA negative control; si-lncUSMycN, siRNA lncUSMycN

To study apoptosis by focusing on morphological changes, siRNA-treated and control cells were stained with acridine orange/ethidium bromide. As shown in Figure 3A, the knockdown of lncUSMycN induces apoptosis-related morphological changes. Consistent with the morphological changes, the gene expression data revealed that lncUSMycN knockdown significantly up-regulated Bax expression (*p *= 0.015) and down-regulated Bcl-2 at the mRNA level in ZR-75-1 cells (*p *= 0.041, Fig. 3B).

**Table 2:** The relationship between lncUSMycN expression levels and clinicopathological features of patients with breast cancer

**Characteristics**	**No. of** **patients (%)**	**lncUSMycN** **ΔCt mean ± SEM**	***p*** **value**
Age <45 >45	24 (46.7) 28 (53.3)	18.11 ± 0.86 17.13 ± 0.70	0.379
Tumor Size <2 cm >2 cm	24 (46.2) 20 (38.5)	16.34 ± 0.72 18.04 ± 0.92	0.148
TNM* clinical stage I II III	17 (32.7) 14 (26.9) 14 (26.9)	15.80 ± 0.92 16.43 ± 1.06 20.28 ± 0.88	**0.004** **
Lymphatic methastasis Absent Present	17 (32.7) 28 (53.8)	15.79 ± 0.92 18.11 ± 0.72	0.052
Differentiation Poor Moderate Well	5 (9.6) 35 (67.3) 5 (9.6)	14.16 ± 1.53 17.58 ± 0.63 17.91 ± 2.31	0.176
Progestron expression (%) <30	18 (34.6) 16 (30.8)	17.54 ± 0.95 16.31 ± 1.02	0.38
Estrogen expression (%) <30 >30	17 (32.7) 17 (32.7)	18.16 ± 0.81 15.77 ± 1.07	0.085
HER-2 status Negative Positive	16 (30.8) 15 (28.8)	16.86 ± 1.04 17.14 ± 1.10	0.858
Location Right Left	20 (38.5) 23 (44.2)	16.68 ± 0.88 18.12 ± 0.73	0.214

** The value in bold is significant (*p *<0.01)

**LncUSMycN knockdown affected ZR-75-1** **cell cycle distribution **

**Fig. 4:**
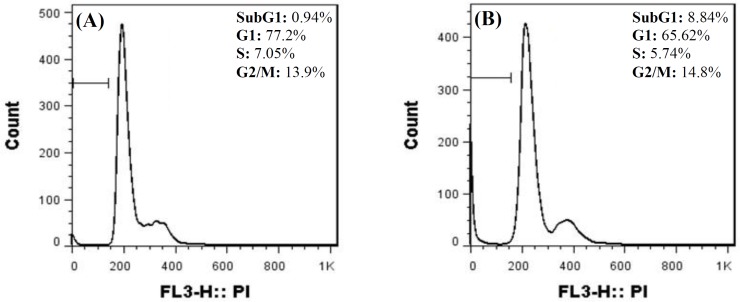
ZR-75-1 cells were transfected with si-NC (A) and si-lncUSMycN (B). PI staining was done 72 h after transfection. The range gate illustrated on FACS plots indicates subG1. si-NC, siRNA negative control; si-lncUSMycN, siRNA lncUSMycN; PI, propidium iodide

Flow cytometry was applied to study the growth inhibitory effect of lncUSMycN knockdown on ZR-75-1 cells. Our data showed that lncUSMycN knockdown influenced ZR-75-1 cell population in subG1 and G1 phases. The results for subG1 and G1 were 0.94% and 77.2%, respectively. Nevertheless, siRNA-transfected cell population for subG1 and G1 was reported as 8.84% and 65.6%, respectively. Therefore, cell cycle analysis showed a significant reduction in G1 phase (*p *< 0.05), while the subG1 population, representing apoptotic cells, was significantly increased 72 h after si-lncUSMycN transfection (*p* < 0.05, Fig. 4).

**lncUSMycN knockdown affects epithelial-mesenchymal transition** (**EMT) **

**Fig. 5:**
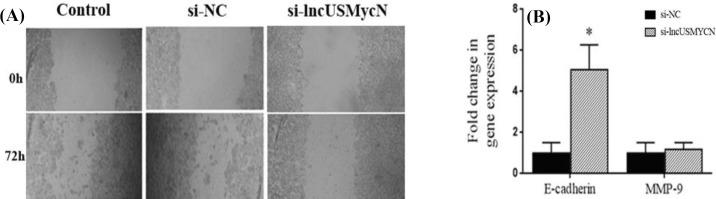
Effects of lncUSMycN knockdown on epithelial-mesenchymal transition . (A) Scratch wound-healing assay was performed in si-NC and si-lncUSMycN transfected breast cancer cell line ZR-75-1 (magnification 100 ); (B) analysis of E-cadhein and MMP-9 expression in ZR-75-1 cells by qRT-PCR 72 h after transfection (^*^p < 0.05). si-NC, siRNA negative control; si-lncUSMycN, siRNA lncUSMycN

To explore the role of lncUSMycN in invasion and EMT, the invasive behavior of ZR-75-1 cells following depletion of lncUSMycN was examined. The effect of lncUSMycN on migration was determined by wound healing assay. The results revealed that the knockdown of lncUSMycN significantly decreased the cell migration capacity as compared to non-transfected parental cells or si-NC-treated cells (Fig. 5A). To further investigate the role of lncUSMycN in invasion and metastasis of breast cancer, the expression of E-cadherin, as a EMT marker, and MMP-9 was evaluated. Consistent with the wound healing assay results, depletion of lncUSMycN significantly up-regulated E-cadherin in ZR-75-1 cells (*p *= 0.025) but had no significant effect on MMP-9 expression (*p *> 0.05, Fig. 5B).

## DISCUSSION

Given that breast cancer is the most common cancer among women worldwide^[^^8^^]^ and the crucial role of lncRNAs in cancer initiation and progression^[^^9^^]^, the present study was undertaken to examine the expression levels of lncRNA lncUSMycN as well as its cellular role in breast cancer. In concordance with Liu *et al.*'s^[^^7^^]^ findings, our results revealed the elevated expression levels of lncUSMycN in breast cancer tissues compared to the adjacent non-tumor specimens. The potential value of this lncRNA for early diagnosis of breast cancer was also demonstrated. In addition, cellular function of lncUSMycN was investigated in a cell model to realize its possible role in breast cancer. Attenuation of lncUSMycN was found to result in apoptotic morphological changes and overexpression of EMT marker (E-cadherin) in breast cancer cells.

Inducing apoptosis is an important approach to controlling tumor development. Bcl-2 protein family members such as anti-apoptotic Bcl-2 and pro-apoptotic Bax are the key elements in apoptosis^[^^10^^,^^11^^]^, which make them promising targets in cancer therapy^[^^12^^,^^13^^]^. 

In this study, we showed that lncUSMycN knockdown probably induced apoptosis through the suppression of Bcl-2 and overexpression of Bax in breast cancer cells. Furthermore, we found that lncUSMycN depletion resulted in significantly lower expression of Bcl-2 and higher expression of E-cadherin in breast cancer cells. In addition, lncUSMycN knockdown significantly suppressed cellular migration. However, lncUSMycN silencing had no significant effect on MMP-9 expression. Our results are consistent with previous studies in which the up-regulation of Bcl-2 led to the loss of E-cadherin and decreased cadherin-mediated cell-cell adhesion as well as invasion^[^^14^^-^^16^^]^. Hence, our findings further emphasize the critical role of E-cadherin in EMT, which is an important step in metastasis^[17,18]^. Another possible explanation by which lncUSMycN could affect E-cadherin levels might be related to the role of miR-9 in EMT^[^^19^^]^. Ma *et al.*^[^^19^^] ^have demonstrated that the activation of pro-metastasis miR-9 by C-Myc and N-Myc oncoproteins results in the inhibition of E-cadherin. Given the up-regulation of N-Myc^[^^20^^,^^21^^]^ and miR-9^[^^22^^,^^23^^]^ in breast cancer and increased expression of N-Myc through lncUSMycN^[^^7^^]^, we speculate that the same pathways could be involved in reduced cell invasion after lncUSMycN knockdown. However, further functional studies are required to precisely determine the role of lncUSMycN and its related pathways in cell invasion and apoptosis.

In conclusion, our findings suggest that lncUSMycN is a potential molecular marker for breast cancer and provides further evidence for critical role of this lncRNA in cancer. It also provides preliminary data on the importance of this lncRNA as a possible target therapy against breast malignancy. 

## References

[1] Redig AJ, McAllister SS (2013). Breast cancer as a systemic disease: a view of metastasis. Journal of internal medicine.

[2] American Cancer Society Breast Cancer Facts &amp; Figures 2017-2018.

[3] Esteller M (2011). Non-coding RNAs in human disease. Nature reviews genetics.

[4] Gibb EA, Brown CJ, Lam WL (2011). The functional role of long non-coding RNA in human carcinomas. Molecular cancer.

[5] Batista PJ, Chang HY (2013). Long noncoding RNAs: cellular address codes in development and disease. Cell.

[6] Sun XH, Yang LB, Geng XL, Wang R, Zhang ZC (2015). Increased expression of lncRNA HULC indicates a poor prognosis and promotes cell metastasis in osteosarcoma. International journal of clinical and experimental pathology.

[7] Liu PY, Erriquez D, Marshall GM, Tee AE, Polly P, Wong M, Liu B, Bell JL, Zhang XD, Milazzo G, Cheung BB, Fox A, Swarbrick A, Hüttelmaier S, Kavallaris M, Perini G, Mattick JS, Dinger ME, Liu T (2014). Effects of a novel long noncoding RNA, lncUSMycN, on N-Myc expression and neuroblastoma progression. Journal of the National Cancer Institute.

[8] Jemal A, Bray F, Center MM, Ferlay J, Ward E, Forman D (2011). Global cancer statistics. CA: a cancer journal for clinicians.

[9] Yang G, Lu X, Yuan L (2014). LncRNA: a link between RNA and cancer. Biochemica et biophysica acta.

[10] Gross A, McDonnell JM, Korsmeyer SJ (1999). BCL-2 family members and the mitochondria in apoptosis. Genes and development.

[11] Plati J, Bucur O, Khosravi-Far R (2008). Dysregulation of apoptotic signaling in cancer: molecular mechanisms and therapeutic opportunities. Journal of cellular biochemistry.

[12] Vervloessem T, Kerkhofs M, La Rovere RM, Sneyers F, Parys JB, Bultynck G (2018). Bcl-2 inhibitors as anti-cancer therapeutics: The impact of and on calcium signaling. Cell calcium.

[13] Soderquist RS, Eastman A (2016). BCL2 inhibitors as anticancer drugs: a plethora of misleading BH3 mimetics. Molecular cancer therapeutics.

[14] Li L, Backer J, Wong AS, Schwanke EL, Stewart BG, Pasdar M (2003). Bcl-2 expression decreases cadherin-mediated cell-cell adhesion. Journal of cell science.

[15] Karch I, Schipper E, Christgen H, Kreipe H, Lehmann U, Christgen M (2013). Is upregulation of BCL2 a determinant of tumor development driven by inactivation of CDH1/E-cadherin?. PLoS one.

[16] Sasaki CY, Lin HC, Passaniti A (2000). Expression of E-cadherin reduces Bcl-2 expression and increases sensitivity to etoposide-induced apoptosis. Cancer cell biology.

[17] Thiery JP (2003). Epithelial-mesenchymal transitions in development and pathologies. Current opinion in cell biology.

[18] Myong NH (2012). Loss of E-cadherin and acquisition of vimentin in epithelial-mesenchymal transition are noble indicators of uterine cervix cancer progression. Korean journal of pathology.

[19] Ma L, Young J, Prabhala H, Pan E, Mestdagh P, Muth D, Teruya-Feldstein J, Reinhardt F, Onder TT, Valastyan S, Westermann F, Speleman F, Vandesompele J, Weinberg RA (2010). miR-9, a MYC/MYCN-activated microRNA, regulates E-cadherin and cancer metastasis. Nature cell biology.

[20] Mizukami Y, Nonomura A, Takizawa T, Noguchi M, Michigishi T, Nakamura S, Ishizaki T (1995). N-myc protein expression in human breast carcinoma: prognostic implications. Anticancer research.

[21] Metge BJ, Mitra A, Chen D, Shevde LA, Samant RSJSr (2015). N-Myc and STAT interactor regulates autophagy and chemosensitivity in breast cancer cells. Scientific reports.

[22] Sun Y, Wu J, Wu SH, Thakur A, Bollig A, Huang Y, Liao DJ (2009). Expression profile of microRNAs in c-Myc induced mouse mammary tumors. Breast cancer research and treatment.

[23] Iorio MV, Ferracin M, Liu CG, Veronese A, Spizzo R, Sabbioni S, Magri E, Pedriali M, Fabbri M, Campiglio M, Ménard S, Palazzo JP, Rosenberg A, Musiani P, Volinia S, Nenci I, Calin GA, Querzoli P, Negrini M, Croce CM (2005). MicroRNA gene expression deregulation in human breast cancer. Cancer research.

